# Propensity-score-matched evaluation of the incidence of radiation pneumonitis and secondary cancer risk for breast cancer patients treated with IMRT/VMAT

**DOI:** 10.1038/s41598-017-14145-x

**Published:** 2017-10-23

**Authors:** Pei-Ju Chao, Hsiao-Fei Lee, Jen-Hong Lan, Shih-Sian Guo, Hui-Min Ting, Yu-Jie Huang, Hui-Chun Chen, Tsair-Fwu Lee

**Affiliations:** 1 Medical Physics and Informatics Laboratory of Electronics Engineering, National Kaohsiung University of Applied Sciences, Kaohsiung, 80778 Taiwan ROC,; 2 Department of Radiation Oncology, Kaohsiung Chang Gung Memorial Hospital and Chang Gung University College of Medicine, Kaohsiung, 83342 Taiwan ROC,; 3 Graduate Institute of Clinical Medicine, Kaohsiung Medical University, Kaohsiung 807, Taiwan ROC,; 4 Department of Radiation Oncology, Kaohsiung Yuan’s General Hospital, Kaohsiung, 80249 Taiwan ROC,

## Abstract

Propensity score matching evaluates the treatment incidence of radiation-induced pneumonitis (RP) and secondary cancer risk (SCR) after intensity-modulated radiotherapy (IMRT) and volumetric-modulated arc therapy (VMAT) for breast cancer patients. Of 32 patients treated with IMRT and 58 who received VMAT were propensity matched in a 1:1 ratio. RP and SCR were evaluated as the endpoints of acute and chronic toxicity, respectively. Self-fitted normal tissue complication probability (NTCP) parameter values were used to analyze the risk of RP. SCRs were evaluated using the preferred Schneider’s parameterization risk models. The dosimetric parameter of the ipsilateral lung volume receiving 40 Gy (IV_40_) was selected as the dominant risk factor for the RP NTCP model. The results showed that the risks of RP and NTCP, as well as that of SCR of the ipsilateral lung, were slightly lower than the values in patients treated with VMAT versus IMRT (*p* ≤ 0.01). However, the organ equivalent dose and excess absolute risk values in the contralateral lung and breast were slightly higher with VMAT than with IMRT (*p* ≤ 0.05). When compared to IMRT, VMAT is a rational radiotherapy option for breast cancer patients, based on its reduced potential for inducing secondary malignancies and RP complications.

## Introduction

Intensity-modulated radiotherapy (IMRT) and volumetric-modulated arc therapy (VMAT) are two of the main therapeutic modalities for patients with breast cancer^1^. However, the potential complications associated with these treatments include acute and chronic toxicity. In acute toxicity, radiation-induced pneumonitis (RP) is the major side effect involving the lung after radiotherapy (RT), and it reduces patient quality of life (QoL)^2,3^. The risk of RP toxicity is evaluated using normal tissue complication probability (NTCP) models. In chronic toxicity, the secondary cancer risk (SCR) must be taken into consideration, especially in younger women with early stage breast cancer as they may go on to live a normal life^4,5^. Schneider *et al*. proposed several models to effectively evaluate the SCR^6,7^.

To date, no randomized trial has been conducted in breast cancer patients simultaneously comparing the treatment-related incidence of RP and the effects of SCR after IMRT/VMAT^8^. Non-randomized comparisons may be limited by two sets of baseline features with a disequilibrium distribution^9^. In general, the presence of confounders may favor one therapeutic technique over the other, whereas the removal of confounding effects will allow an unbiased analysis and an estimate of the treatment effect. In practice, confounders can be eliminated using propensity score-matched analysis,^9,10^ which allows data matching between general baseline factors to establish two similar datasets for comparison. Therefore, in this study, a confounder-elimination process was conducted using propensity score matching to compare the incidence of radiation-induced RP and chronic SCR toxicity for breast cancer patients treated with IMRT/VMAT.

## Methods and Materials

### Patient characteristics

**Figure 1:**
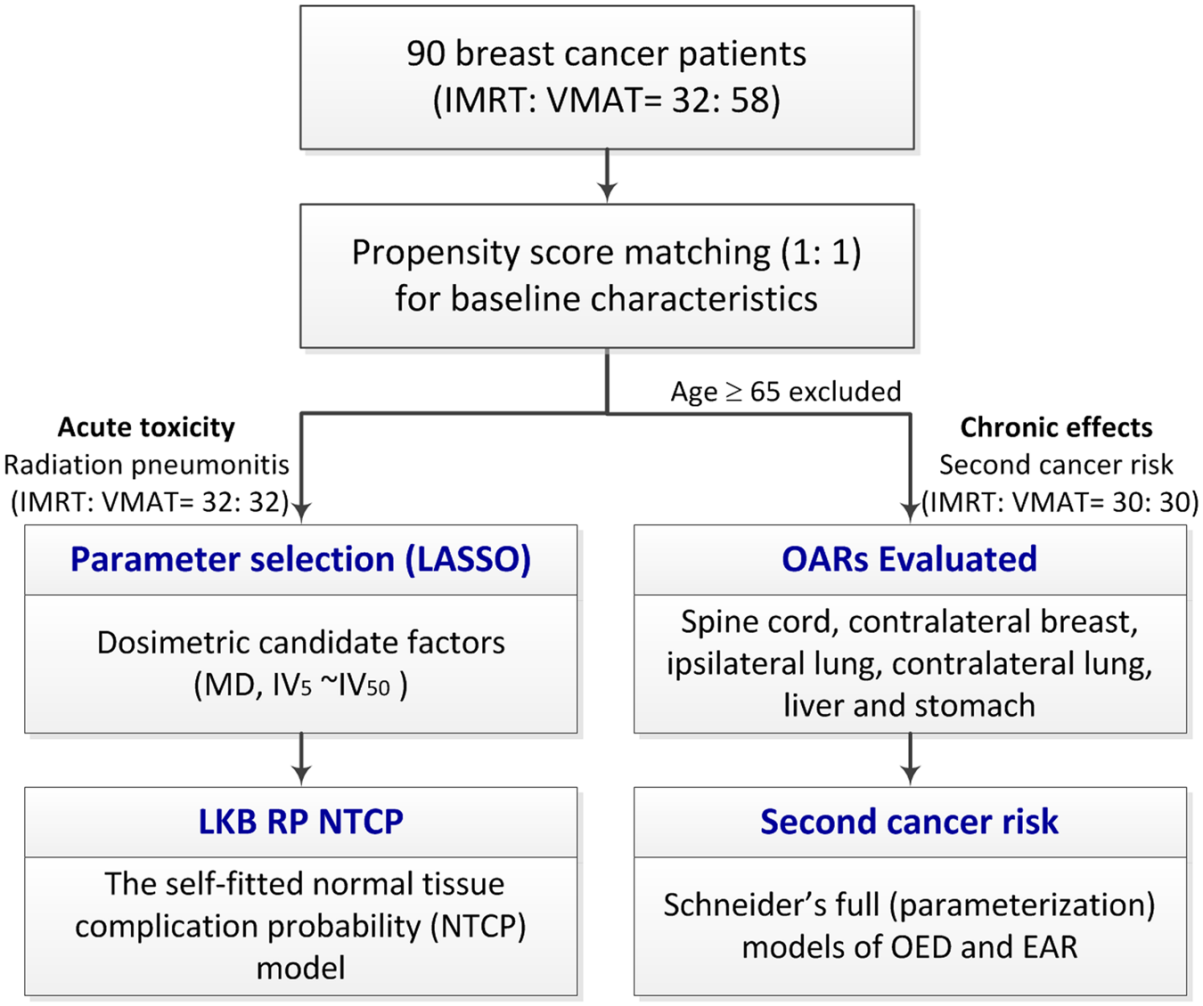
The process profile for this study. *Abbreviations*: LASSO, least absolute shrinkage and selection operator; IMRT, intensity-modulated radiotherapy; VMAT, volumetric-modulated arc therapy; RP, radiation-induced pneumonitis; IV_5_: the volume of the ipsilateral lung receiving at least 5 Gy; the following parameters are similar; IV_10_∼IV_50_; NTCP, normal tissue complication probability; MD, mean dose to the ipsilateral lung; LKB, Lyman-Kutcher-Burman; OED, organ equivalent dose; EAR, excess absolute risks; OARs, organs at risk.

A total of 90 breast cancer patients were enrolled. All of the patients were treated between January 2012 and December 2015. Thirty-two patients were treated with IMRT and 58 with VMAT. The patients’ baseline characteristics were recorded, including basic information, disease status, and RT parameters (Table 1). All experimental protocols of this study were approved by the Institutional Review Board of Chang Gung Memorial Hospital (104-8255B). Figure 1 shows the process profile; in terms of the radiation-induced parameters, patients were propensity-score-matched in a 1:1 ratio according to their age at treatment, American Joint Committee on Cancer (AJCC) stage, irradiation to supraclavicular fossa (SCF) status, and concurrent chemotherapy. Therefore, the confounders derived from the baseline characteristics could be eliminated by propensity score matching. RP was evaluated as the endpoint of acute toxicity and SCR as the endpoint of chronic toxicity. Propensity score matching was performed using a caliper distance of 0.2 (i.e., 0.2 × the standard deviation of the logit of the propensity score) without replacement, as recommended^11,12^. Data matching was performed using SPSS software (ver. 22.0; IBM SPSS, Chicago, IL, USA).

**Table 1:** Unmatched and propensity score-matched baseline characteristics.

Patient characteristics		Unmatched Cohort			Propensity Matched Cohort		
		IMRT *n* = 32 (%)	VMAT *n* = 58 (%)	*p*	IMRT *n* = 32 (%)	VMAT *n* = 32 (%)	*p*
Age	Mean	53	50	0.47	53	51	0.88
	Range	28–70	33–75		28–70	33–74	
	≦40	3 (9)	10 (17)		3 (9)	4 (12)	
	41–50	8 (25)	16 (28)		8 (25)	9 (28)	
	51–60	14 (44)	19 (33)		14 (44)	12 (38)	
	>61	7 (22)	13 (22)		7 (22)	7 (22)	
SCF	NO	13 (41)	40 (69)	0.60	13 (41)	21 (66)	0.60
	Yes	19 (59)	18 (31)		19 (59)	11 (34)	
AJCC stage	1	16 (50)	37 (64)	0.03	16 (50)	16 (50)	1.00
	2	5 (16)	10 (17)		5 (16)	5 (16)	
	3	11 (34)	11 (17)		11 (34)	11 (34)	
T stage	1	12 (38)	32 (56)	0.53	12 (38)	15 (47)	0.77
	2	18 (56)	22 (38)		18 (56)	14 (44)	
	3	2 (6)	2 (3)		2 (6)	2 (6)	
	4	0	2 (3)		0	1 (3)	
N stage	0	14 (44)	37 (64)	0.13	14 (44)	17 (53)	0.94
	1	8 (25)	12 (21)		8 (25)	6 (19)	
	2	4 (13)	5 (9)		4 (13)	5 (15)	
	3	6 (19)	4 (6)		6 (19)	4 (13)	
Chemotherapy	NO	10 (31)	29 (50)	0.67	10 (31)	16 (50)	0.67
	Yes	22 (69)	29 (50)		22 (69)	16 (50)	
RP	Grade 0	14 (44)	29 (50)		14 (44)	19 (59)	
	Grade 1	10 (31)	20 (34)		10 (31)	10 (31)	
	Grade 2	7 (22)	7 (11)		7 (22)	2 (6)	
	Grade 3	1 (3)	2 (4)		1 (3)	1 (3)	

*Abbreviation*: IMRT: intensity modulated radiotherapy; VMAT: volumetric modulated arc therapy; SCF: irradiation to supraclavicular fossa; AJCC American Joint Committee on Cancer; RP: radiation-induced pneumonitis.

### Treatment techniques

The details of the treatment techniques were reported previously^2^. Philips Pinnacle^3^ treatment planning system (TPS) (V 9.2; Philips, Fitchburg, WI, USA) was used for planning. Treatment was delivered using an Elekta Precise linear accelerator (Elekta, Crawley, UK) or a Varian Clinac IX (Varian Medical Systems, Palo Alto, CA, USA). Patients received either IMRT or VMAT with a simultaneous integrated boost technique (SIB) and a single 6-MV energy setting. IMRT patient treatments were planned with a six/seven-field plan. VMAT used a five/six-partial-arc plan by incorporating arc angle designs and the field size opening to reduce exposure to the ipsilateral lung and contralateral breast. The details of the IMRT protocol, including the prescribed dosage and fractions for the SIB technique, were reported previously^13,14^, as were those of the multiple partial VMAT technique^15^. The prescribed doses were 63 Gy to planning target volume 1 (PTV1) (macroscopic tumor) and 51 Gy to PTV2 (subclinical disease area). They were delivered at 1.7–2.1 Gy per fraction using SIB with five fractions per week. The dosimetric data were obtained from the dose-volume histograms (DVH) on the Pinnacle^3^ TPS with a bin size and dose calculation resolution of 1 cGy and 2.5 mm, respectively. Figure 1a,b shows the planning computed tomography (CT) scans of two representative patients showing the calculated isodoses for IMRT and VMAT. The decision regarding the need for adjuvant chemotherapy included consideration of the risk of recurrence, toxicity, and comorbidities. The schedule and regimens were modified according to the patient’s clinical condition and the oncologist’s judgment, as necessary.

#### Evaluation of RP

The final endpoint of acute RP was described in a previous study^2^. In this study, acute RP was defined based on chest CT images as density changes ≥ grade 1 (clinically symptomatic pneumonitis was not included because the radiographic findings were very similar to those of pneumonitis symptoms^2^). Chest CT images were analyzed 1–3 months after RT completion. Evaluation of the chest CT density changes was based on a comparison with the CT image taken prior to RT. Arriagada’s classification^16,17^ was used to grade the density changes for RP: complete opacity, 3; moderate opacity, 2; low opacity in linear streaks, 1; no change, 0.

#### RP NTCP modeling

After propensity score-matching, 10 dosimetric candidate risk factors were selected as the initial variables (Supplementary Table S1), including the MD (mean dose), the IV_5_ (the volume of the ipsilateral lung receiving at least 5 Gy; other parameters were similar), and the IV_10_~IV_50_. The least absolute shrinkage and selection operator (LASSO) was used to select the best potential predictive dosimetric factor for RP occurrence. The online Matlab package (LASSOGLM) was used to fit the process of LASSO. Thus, the LASSO technique was performed in the context of 10-fold cross validation; the parameters (alpha and lambda values) were based on those of previous studies^18–20^. Alpha was set to 1, and the lambda value was set to the default setting (minimal mean squared error plus 1 standard deviation). This regularization technique identifies the most important factor for RP occurrence. The most significant dosimetric factor thus identified was used to develop a single Lyman-Kutcher-Burman (LKB) NTCP model for RP complication sparing. The TV_50_ and m in the LKB NTCP model were determined by curve fitting using a maximum likelihood analysis, with n set to 1. Both the LASSO technique and the LKB NTCP analysis were previously described^2,18,21,22^. After the LKB NTCP predictive model was created, the area under the curve (AUC), calibration slope, scaled Brier score, and Hosmer-Lemeshow tests were used to verify the system performance.

#### Evaluation of the excess absolute risk of carcinoma induction

Schneider’s full (parameterization) models of organ equivalent dose (OED) and excess absolute risk (EAR) were used for the carcinoma induction analyses. The OED and EAR concepts were previously reported^23,24^. The risk equivalent dose (RED) is a dose-response-weighted tissue dose value that is proportional to the risks. RED is averaged over the OED; the valve is expressed in Gy and is proportional to the developing cancer risk in the organ. The impact of fractionation, given by α′ = α + βD, α/β = 3 Gy, was used in this study. RED was also used to evaluate the EAR, as described previously^23,24^. Thus, the risk of EAR in an organ with volume V_T_ at one age (age_x_) following exposure to a RED and re-exposure of the patient at an older age (age_a_) was defined, in which parameters *γ*_*e*_ and *γ*_*a*_ are the modifying factors for age and β was defined for people exposed at age 30 years and again at age 70 years. The SCR was evaluated for the spinal cord, contralateral breast (C-Breast), contralateral lung (C-Lung), ipsilateral lung (I-Lung), liver, and stomach, all of which were assigned calculation parameter values taken from Schneider’s full risk models. The EAR was calculated for people from the exposed age to the age of 70 years. The parameters used for Schneider’s OED and EAR models are listed in Supplementary Table S2. The parameters used for β (as excess cases per 10,000 person-years [PY]/Gy), γ_e_, γ_a_, and R were taken from a previous study^24^.

## Results

**Figure 2:**
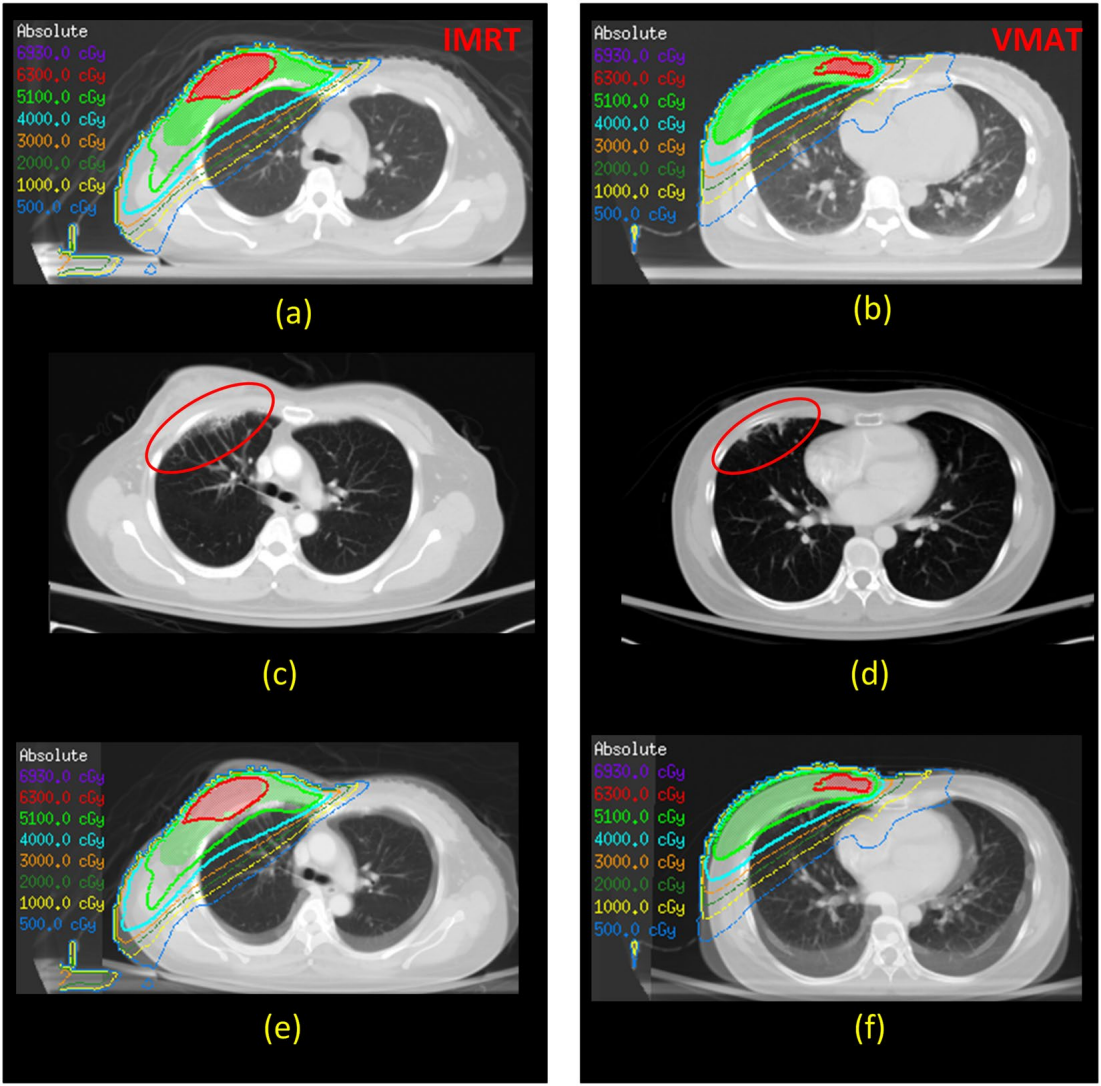
Two representative treatment plans (**a**,**b**); RP-diagnosed at 3 months after RT (**c**,**d**); RP-diagnosed images merged with the original isodose curves (**e**,**f**). *Abbreviations*: RT, radiotherapy; RP, radiation pneumonitis; IMRT, intensity-modulated radiotherapy; VMAT, volumetric-modulated arc therapy.

In the unmatched population, patients treated with VMAT were younger and had a higher AJCC stage distribution (*p* ≤ 0.05). Patients receiving concurrent chemotherapy had a non-significant difference compared to patients receiving RT alone (*p* ≥ 0.6). The study cohort characteristics were stratify-matched according to AJCC stage and the patient’s age. The treatment plans of two representative patients are shown in Fig. 2a,b; images from a patient diagnosed with RP 3 months after RT are shown in Fig. 2c,d. Images from a patient diagnosed with RP and merged with the original isodose curves are shown in Fig. 2e,f.

**Figure 3:**
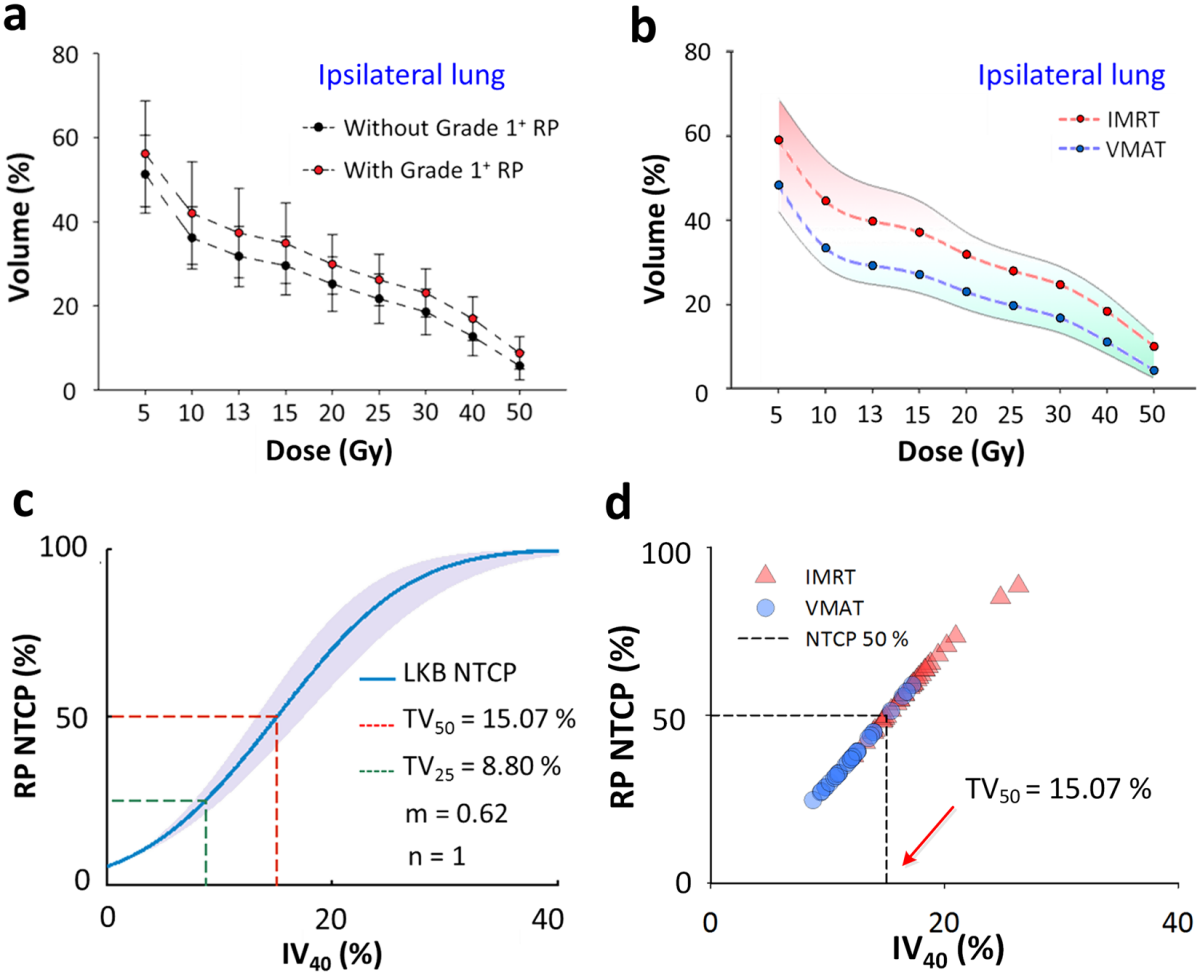
Average dose volume histograms for the mean doses delivered to the ipsilateral lung in the groups (**a**) with/without RP and (**b**) the IMRT/VMAT, respectively. The self-fitted dose-response LKB RP NTCP curve (**c**). Scatter plots for the IV_40_ and NTCP between the IMRT and VMAT cohorts (**d**). *Abbreviations*: IMRT, intensity-modulated radiotherapy; VMAT, volumetric-modulated arc therapy; RP, radiation-induced pneumonitis; IV_40_: the volume of the ipsilateral lung receiving at least 40 Gy; the following parameters are similar; IV_5_∼IV_50_; DF, degrees of freedom; MD, mean dose to the ipsilateral lung; NTCP, normal tissue complication probability; LKB, Lyman-Kutcher-Burman; TD_25_, the dose predicting a 25% risk of complications; n, a parameter that considers the volume effect; m, a dimensionless model parameter for describing the slope of the dose-response curve.

The two groups, with a total of 64 matched patients (32:32), did not differ significantly (all *p* ≥ 0.6; Table 1). After RT, of the 64 matched patients, 33 (52%), 20 (31%), 9 (14%), and 2 (3%) patients had density changes on their chest CT images of grade 0, 1, 2, and 3, respectively. RP was diagnosed in 48% (31/64) of the patients. The average DVHs of the MDs received by the ipsilateral lung in the group with/without RP occurrence, and in the IMRT/VMAT groups, are shown in Fig. 3a,b, respectively.

**Figure 4:**
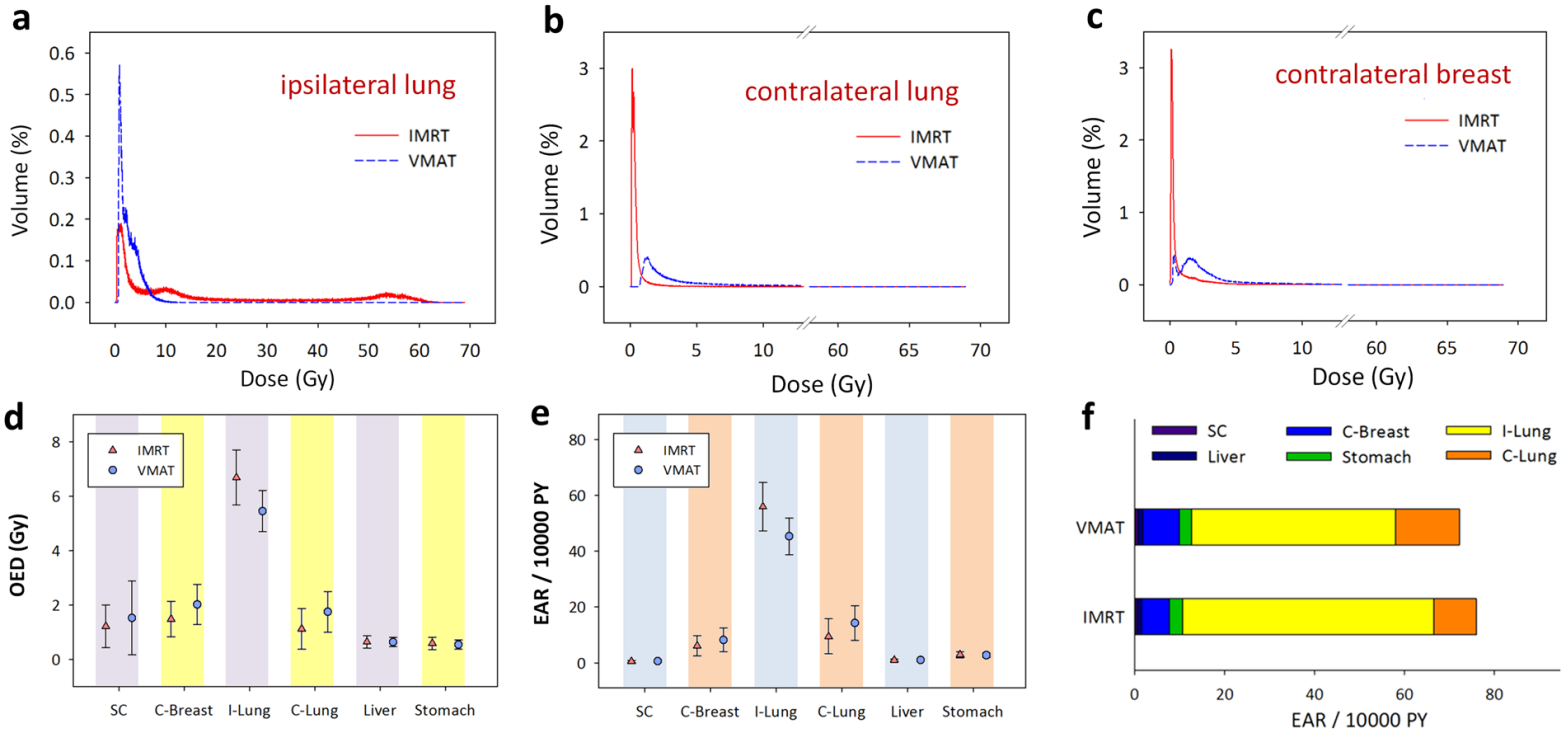
Differential dose volume histograms of the same two representative patients for irradiation of the ipsilateral and contralateral lung and contralateral breast for IMRT/VMAT breast radiotherapy (**a**,**b**,**c**). OED/EAR of 30 patients (mean and standard deviation) using Schneider’s full model for 6 OARs for IMRT and VMAT (**d**,**e**). Accumulated EARs for 6 OARs at age of 70 years (**f**). The EAR has units of excess cases per 10,000 person-years (PY)/Gy. *Abbreviations*: IMRT, intensity-modulated radiotherapy; VMAT, volumetric-modulated arc therapy; OED, organ equivalent dose; EAR, excess absolute risks; SC, spinal cord; C-Breast, contralateral breast; I-Lung, ipsilateral lung; C-Lung, contralateral lung; OARs, organs at risk.

The dosimetric parameter ipsilateral lung volume receiving ≥ 40 Gy (IV_40_) was selected as the dominant risk factor for the RP NTCP model by LASSO using cross-validation. The LASSO trace plot of dosimetric candidate predictive factors and the factor ranking list are provided in Supplementary Figure S1. The cross-validated mean squared error (MSE) of the LASSOGLM fit is shown in Supplementary Figure S2. The self-fitted dose-response LKB RP NTCP curve (using IV_40_) for the incidence of grade 1+ RP is shown in Fig. 3c. Therefore, we determined the tolerance of IV_40_ producing a 50% complication rate (TV_50_) to be 15.07% (95% confidence interval [95% CI]: 13.21–17.49) and the slope (m) was equal to 0.62 (95% CI: 0.42–1.08). The overall performance for the IV_40_-fitted NTCP model tested using the AUC, the Hosmer–Lemeshow test, scaled Brier score, and calibration slope was 0.72, 0.82, 0.43, and 0.96, respectively. A total of 31 events (with grade 1+ RP) were observed; 18 in the IMRT group and 13 in the VMAT group. As a result, patients with RP were less common in the VMAT group than those treated with IMRT (*p* = 0.04). Scatter plots for IV_40_ and NTCP are shown in Fig. 3d. The absorbed doses for six OARs are given in Supplementary Table S3. Patients aged more than 65 years were excluded from the SCR analysis, while 60 patients were enrolled (30: 30 for IMRT: VMAT). Figure 4(a–c) shows the differential DVHs of the same two representative patients for irradiation of the ipsilateral and contralateral lung and contralateral breast for IMRT/VMAT. Differences in the dose distributions are visible, particularly in the low-dose regions in the OARs.

**Figure 5:**
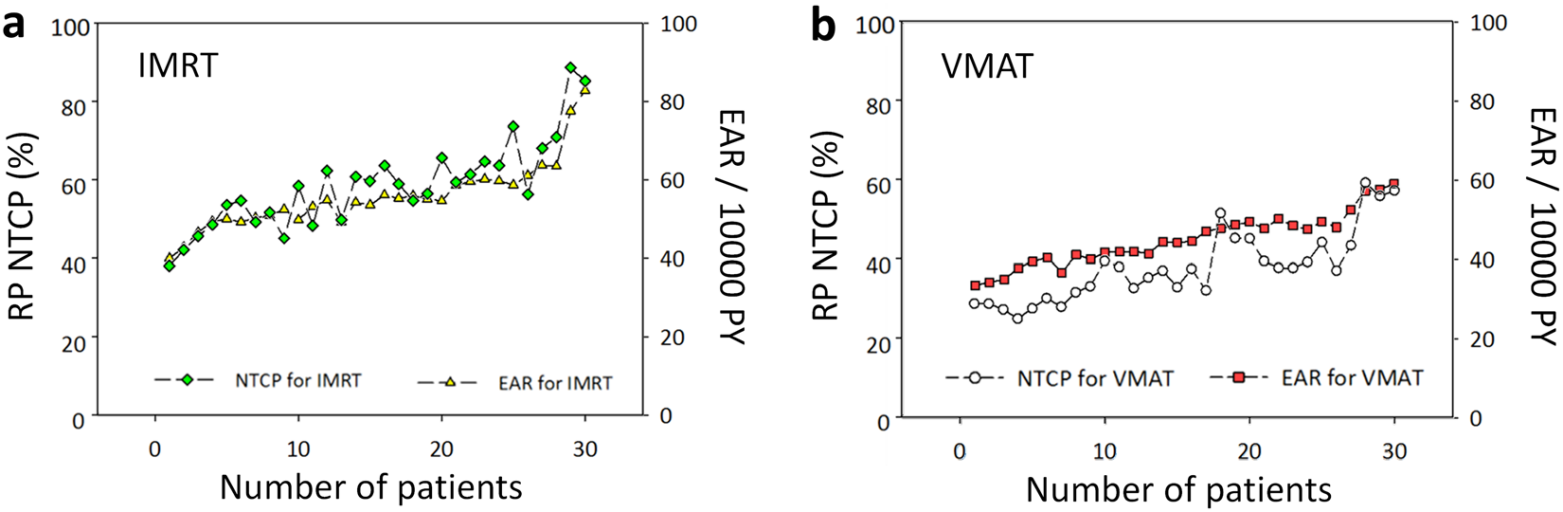
The trends between RP NTCP and EAR of ipsilateral lung for patients treated with (**a**) IMRT or (**b**) VMAT. The EAR has units of excess cases per 10,000 person-years (PY)/Gy. *Abbreviations*: IMRT, intensity-modulated radiotherapy; VMAT, volumetric-modulated arc therapy; NTCP, normal tissue complication probability; EAR, excess absolute risk.

Figure 4(d,e) shows the differences in OED/EAR for six OARs using the Schneider parameterization dose-response model between patients treated with IMRT and VMAT. Figure 4f shows accumulated EARs for six OARs. VMAT only showed a significantly lower OED and EAR compared to IMRT of the ipsilateral lung (*p*≤ 0.01). However, the values of OED and EAR in the contralateral lung and breast were slightly higher in VMAT than in IMRT patients (*p* ≤ 0.05). Details for OED/EAR values can be found in Supplementary Tables S4–S6. Figure 5a and b present the trends between RP NTCP and EAR. The risk for RP NTCP and EAR on VMAT were both slightly lower than that of patients treated with IMRT.

## Discussion

Propensity scoring methods allow researchers to evaluate the treatment-related factors implicated in illness using observational or nonrandomized data^25^. The results of the propensity score analyses done for this study were compared to our previous results, obtained using a LASSO NTCP model processing^2^. Without propensity-score matching, confounders such as age at treatment, energy used, and body mass index (BMI) may lead to a biased and localization-suitable result. Previous studies^2,26,27^ showed that IV_20_ and age correlated significantly and positively with the incidence of symptomatic RP, whereas BMI, T-stage, and energy setting correlated negatively, but not significantly, with the incidence of symptomatic RP. Estimation of the radiation-induced effects of treatment on outcomes with propensity score matching is recommended to determine factors that may affect the results. When the baseline treatment-related characteristics of the patients (Table 1, all *p* ≥ 0.6), except for dosimetric factors, were controlled by the matching process, the dosimetric factors could be identified without bias, allowing the assessment of their relevance. After propensity score matching using the LASSO feature selection, IV_40_ (volume) was identified as the most significant predictive factor for the LKB RP NTCP model. To keep the incidence of RP under 50% (TV_50_) after NTCP curve fitting, the IV_40_ volume needs to be <15.07% in patients treated with IMRT/VMAT. Thus, to maintain the incidence of grade RP at <25%, our results suggest that the IV_40_ volume should be <8.8%.

RP matching analysis for the IMRT group versus the VMAT group subsets was analyzed for 18 of 32 and 13 of 32 incidences, respectively. RP toxicity was significantly improved in patients who underwent VMAT treatment compared to those treated by IMRT. In their analysis of the risk of radiation-induced secondary cancer, Paganetti *et al*.^28,29^ reported that the treatment of a smaller volume with a higher dose is better than the treatment of a larger volume with, on average, a lower dose. However, the development of other side effects also needs to be taken into account. In our study, RP should be taken into consideration to ensure a better quality of life and compliance for breast patients, and compromises should be made carefully while the treatment strategy is undertaken.

Tsai *et al*. compared VMAT to current treatment techniques, such as fixed-beam IMRT and tomotherapy. They found that VMAT strategies resulted in a reduced low-bath dose to the lung area^15^. In the current study, VMAT generally exposed smaller volumes of ipsilateral lung to low-dose regions than was the case with IMRT, but the exposed volumes in the contralateral lung and breast were larger. The MD to the ipsilateral lung was lower in VMAT than in IMRT but the evaluated risk of secondary cancer in the contralateral lung and breast in patients treated with VMAT was slightly higher. On average, IMRT plans carried an approximately 5% higher overall secondary cancer risk than VMAT plans. The greatest discrepancy involved the ipsilateral lung (55.90/10,000 PY on IMRT vs. 45.35/10,000 PY on VMAT). Similar findings have been reported in other studies^30^, including for breast cancer risk, by Hancock *et al*.^31^ (EAR Breast = 21.5/10,000 PY), Dores *et al*.^32^ (10.5/10,000 PY), Swerdlow *et al*.^33^ (3.1/10,000 PY), and van Leeuwen *et al*.^34^ (29.4/10,000 PY). Our results on the contralateral breast were similar: 6.05/10,000 PY on IMRT and 8.14/10,000 PY on VMAT.

Howlader *et al*.^35^ showed that breast cancer is often diagnosed in younger patients (a median age of 61 years) than is the case for colorectal cancer (69 years) and lung cancer (70 years). In this study, approximately 60% of the patients diagnosed with breast cancer were younger than 60 years of age. Thus, more attention should be paid to younger patients in clinical practice. Meadows *et al*.^36^ performed a large investigation of pediatric cancer survivors and showed that almost 10% suffered a secondary neoplasm over a 30-year period after the first diagnosis was confirmed; the majority of the affected patients were females with breast tumors. As pointed out by Paganetti^37^, the reduction of the average age of RT patients and the emergence of more complex treatment regimens will raise concern regarding the incidence of radiation-induced secondary cancers.

Our study was limited by the small number of patients. To improve the performance of the current risk models and decrease the uncertainties in the model parameters, longer and larger epidemiological studies are needed.

Regarding chemotherapy in this study, chemotherapy plus radiotherapy were well tolerated by patients in both groups with good treatment compliance. However, whether the additional chemotherapy had any effect in terms of RP or secondary cancer induction remains unknown.

## Conclusions

VMAT achieved satisfactory planned target dose coverage while maintaining low doses to OARs, thereby reducing the potential for the induction of secondary malignancies and RP complications. VMAT is a reasonable treatment option for breast cancer patients, allowing greater treatment compliance and a better quality of life in the future.

### Data availability

Due to ethical and legal restrictions, data for this manuscript is available under a formal request to the corresponding author and the Chang Gung Memorial Hospital Institutional Review Board.

### Ethical approval and informed consent

All experimental protocols of this study were approved by the Institutional Review Board of Chang Gung Memorial Hospital (104-8255B); the patient informed consent was waived by the institutional review board; and all experiments were performed in accordance with relevant international and national guidelines and regulations.

## Electronic supplementary material

Supplementary Information
