# Supplementary material for: Propensity-score-matched evaluation of the incidence of radiation pneumonitis and secondary cancer risk for breast cancer patients treated with IMRT/VMAT
Source: Sci Rep. 2017 Oct 23;7:13771. doi: 10.1038/s41598-017-14145-x (PMC5653804; doi:10.1038/s41598-017-14145-x)
Supplement: Supplementary file 1 — Supplementary Information [file 41598_2017_14145_MOESM1_ESM.pdf]

Propensity score-matched evaluation of the incidence of radiation pneumonitis and secondary cancer risk for breast cancer patients treated with IMRT/VMAT

Pei-Ju Chao, PhD[1,2], Hsiao-Fei Lee, MS[1,2], Jen-Hong Lan, MS[1,2], Shih-Sian Guo, MS[1,2], Hui-Min Ting, PhD[1,2], Yu-Jie Huang, MD PhD[2], Hui-Chun Chen, MD[2,*], Tsair-Fwu Lee, PhD[1,2,3,4,*],

[1] Medical Physics and Informatics Laboratory of Electronics Engineering, National Kaohsiung University of Applied Sciences, Kaohsiung 80778, Taiwan, ROC

[2] Department of Radiation Oncology, Kaohsiung Chang Gung Memorial Hospital and Chang Gung University College of Medicine, Kaohsiung 83342, Taiwan, ROC

[3] Graduate Institute of Clinical Medicine, Kaohsiung Medical University, Kaohsiung 807, Taiwan, ROC

[4]Department of Radiation Oncology, Kaohsiung Yuan's General Hospital, Kaohsiung 80249, Taiwan, ROC.

Supplementary

Tables

Table S1. Initial candidate predictive factors for RP NTCP modeling.

No.	Description	Range or Classification	Median or frequency	*p*-valve
1	MD	8.75-26.34	14.99	0.009
2	IV_{5}	33.48-91.75	53.67	0.095
3	IV_{10}	21.99-82.80	38.99	0.039
4	IV_{13}	18.70-74.53	34.48	0.027
5	IV_{15}	17.14-68.30	32.12	0.022
6	IV_{20}	14.23-48.09	27.44	0.013
7	IV_{25}	11.56-40.45	23.84	0.008
8	IV_{30}	9.15-37.50	20.73	0.005
9	IV_{40}	4.90-31.58	14.73	0.003
10	IV_{50}	0.87-16.46	7.19	0.004

Abbreviations: MD, mean dose to the ipsilateral lung; IV5 (the volume of the ipsilateral lung receiving at least 5 Gy; the following parameters are similar), IV10~IV50; *p*-valve, univariate logistic test.

Table S2. Parameters used for Schneider's parameterization models

Organs	α	R	β	γ_e	γ_a
BS and SC	0.018	0.23	0.7	-0.024	2.38
Breast	0.044	0.15	8.2	-0.037	1.70
Lung (total)	0.042	0.83	8.0	0.002	4.23
Liver	0.323	0.29	2.4	-0.021	3.60
Stomach	0.460	0.46	5.2	-0.002	1.90

Abbreviation: Note this β is used for EAR calculation only. β within the α/β ratio is calculated from α based on α/β = 3 Gy for all tissues. The EAR has units of excess cases per 10,000 person-years (PY)/Gy.

Table S3. Absorbed dose results for 6 OARs in patients treated with IMRT/VMAT

	IMRT							VMAT						
	Mean dose (Gy)						IV_{40} (%)	Mean dose						IV_{40} (%)
No.	SC	Liver	C-Breast	Stomach	C-Lung	I-Lung	I-Lung	SC	Liver	C-Breast	Stomach	C-Lung	I-Lung	I-Lung
1	0.18	0.44	0.90	5.31	0.64	12.22	12.54	0.46	1.45	1.41	0.72	2.52	9.82	16.48
2	0.19	0.65	0.59	4.30	0.29	13.21	14.79	0.46	1.45	1.41	0.72	0.92	9.82	9.47
3	0.25	3.69	2.29	0.38	0.51	14.06	15.33	0.52	0.53	2.29	0.51	0.74	9.41	8.19
4	0.25	1.22	3.79	0.88	2.64	14.75	15.51	0.86	2.13	2.68	2.77	2.74	8.75	4.90
5	1.09	0.96	0.78	3.36	0.53	15.92	18.42	1.31	1.71	2.96	2.79	1.87	9.48	7.22
6	0.23	3.05	1.11	0.19	0.36	16.16	18.07	0.97	0.84	2.97	0.76	1.06	10.19	7.21
7	2.40	5.06	0.78	0.22	0.52	14.88	14.45	0.44	0.56	1.93	1.44	1.95	10.58	21.00
8	1.54	3.15	2.17	15.67	1.63	15.44	17.43	0.92	1.13	1.45	2.80	1.21	10.98	9.08
9	1.59	7.36	2.46	0.83	0.83	13.91	20.65	0.63	1.23	1.79	0.22	2.07	12.56	14.32
10	1.02	3.59	2.31	0.76	0.73	17.05	18.77	0.87	1.35	2.74	0.88	1.12	12.19	12.01
11	1.81	3.80	9.23	2.39	3.01	14.67	13.98	1.31	2.97	2.07	0.72	1.18	10.85	8.80
12	1.21	3.08	1.41	14.72	0.86	18.00	13.55	1.28	4.10	2.13	1.16	1.27	11.53	10.63
13	1.16	4.91	2.17	4.19	0.56	17.06	17.89	0.95	1.94	2.14	0.66	1.75	11.97	10.68
14	0.41	2.03	1.54	0.25	0.44	17.62	20.74	0.88	0.44	3.20	0.84	1.33	10.92	7.63
15	0.35	2.30	1.39	4.90	0.59	17.35	20.07	1.00	1.25	5.20	0.70	1.40	12.08	9.93
16	0.45	1.96	1.52	2.00	2.82	18.32	22.68	1.86	2.24	2.34	2.18	1.69	12.56	11.79
17	1.46	13.61	2.20	2.38	1.06	17.18	19.37	2.14	0.78	0.86	1.07	1.90	10.71	5.34
18	1.61	9.24	5.93	2.68	1.36	16.17	16.04	0.49	0.96	1.26	3.71	1.13	15.41	8.33
19	0.90	6.90	3.24	0.78	1.80	16.58	17.36	7.79	3.15	2.84	0.55	2.88	13.95	13.13
0	0.31	9.20	1.26	0.26	0.30	18.81	19.97	3.66	1.58	5.16	0.46	2.38	13.93	13.75
21	2.82	0.68	0.59	2.13	0.79	17.30	12.74	1.26	3.95	6.92	2.06	3.87	12.56	8.63
22	1.85	2.77	2.06	7.63	1.07	17.78	18.42	0.99	0.92	2.18	4.65	1.31	12.13	11.64
23	1.60	10.28	1.97	0.77	0.56	18.58	20.49	0.99	0.92	2.18	4.65	1.11	12.13	8.24
24	1.71	0.78	2.73	2.35	1.08	18.33	19.20	0.85	3.18	1.25	0.76	1.01	12.50	8.91
25	4.19	0.50	1.76	3.33	0.93	20.97	24.28	5.22	1.11	3.21	0.62	2.78	13.69	13.17
26	1.91	11.48	5.03	1.09	1.40	16.55	11.30	0.92	1.94	1.61	11.79	1.01	11.98	7.16
27	1.68	3.85	4.11	13.13	2.05	19.46	20.33	1.65	9.96	2.21	1.43	1.50	13.50	9.08
28	2.12	2.50	1.70	9.61	1.04	20.20	23.13	2.94	5.81	2.13	0.40	1.08	17.24	17.13
29	2.24	1.94	2.03	11.18	1.96	26.34	31.58	4.21	1.60	2.15	1.53	2.65	16.43	15.43
30	0.92	6.14	2.66	0.61	0.97	24.81	22.31	2.51	2.18	4.30	4.06	2.00	16.77	15.63

Abbreviations: OED, organ equivalent doses; IMRT, intensity-modulated radiotherapy; VMAT, volumetric-modulated arc therapy; C-Breast, contralateral breast; I-Lung, ipsilateral lung; C-Lung, contralateral lung.

Table S4. OED results for patients treated with IMRT/VMAT

	IMRT - OED (Gy)						VMAT - OED (Gy)					
No.	SC	Liver	C-Breast	Stomach	I-Lung	C-Lung	SC	Liver	C-Breast	Stomach	I-Lung	C-Lung
1	0.18	0.28	0.77	0.49	4.85	0.64	0.60	0.63	1.64	0.53	4.03	2.52
2	0.19	0.29	0.54	0.56	5.03	0.29	0.45	0.60	1.27	0.48	4.11	0.92
3	0.25	0.75	1.18	0.31	5.49	0.51	0.51	0.38	1.74	0.38	4.21	0.74
4	0.25	0.44	2.12	0.49	5.85	2.64	0.85	0.84	2.16	0.83	4.54	2.74
5	1.05	0.35	0.68	0.79	5.92	0.53	1.27	0.52	2.08	0.63	4.76	1.87
6	0.23	0.62	0.81	0.17	5.96	0.36	0.95	0.51	2.31	0.41	4.83	1.06
7	2.11	0.72	0.66	0.19	6.01	0.52	0.43	0.39	1.56	0.57	4.86	1.95
8	1.48	0.99	1.87	0.87	6.20	1.63	0.89	0.57	1.30	0.66	4.98	1.21
9	1.52	0.94	2.01	0.47	6.24	0.83	0.62	0.40	1.51	0.19	5.01	2.07
10	0.98	0.73	1.94	0.45	6.26	0.73	0.85	0.57	2.23	0.34	5.02	1.12
11	1.72	0.81	1.62	0.77	6.35	3.01	1.27	0.76	1.73	0.47	5.06	1.18
12	1.14	0.47	1.14	0.79	6.41	0.86	1.25	0.89	1.83	0.62	5.08	1.27
13	1.15	0.67	1.44	0.62	6.47	0.56	0.93	0.55	1.73	0.40	5.31	1.75
14	0.41	0.61	0.85	0.22	6.49	0.44	0.86	0.35	2.44	0.52	5.39	1.33
15	0.35	0.41	1.03	0.64	6.51	0.59	0.98	0.62	2.87	0.46	5.44	1.40
16	0.44	0.60	1.29	0.72	6.57	2.82	1.62	0.66	1.77	0.58	5.48	1.69
17	1.39	0.93	1.86	0.87	6.60	1.06	2.00	0.53	2.82	0.58	5.73	1.90
18	1.53	0.93	1.89	0.82	6.66	1.36	0.49	0.60	1.12	0.64	5.73	1.13
19	0.88	0.84	2.24	0.53	6.67	1.80	6.39	0.81	2.40	0.40	5.74	2.88
20	0.31	0.80	1.00	0.22	6.69	0.30	3.25	0.61	3.33	0.35	5.80	2.38
21	2.37	0.34	0.51	0.78	6.96	0.79	1.22	0.71	4.57	0.70	5.87	3.87
22	1.74	0.53	1.19	0.85	7.08	1.07	0.97	0.55	1.84	0.72	5.89	1.31
23	1.50	0.92	1.57	0.46	7.18	0.56	0.97	0.55	1.84	0.72	5.89	1.11
24	1.61	0.47	2.20	0.76	7.19	1.08	0.83	0.95	1.16	0.53	5.89	1.01
25	3.44	0.30	1.28	0.80	7.20	0.93	4.41	0.62	2.45	0.43	5.92	2.78
26	1.82	0.92	2.96	0.50	7.22	1.40	0.90	0.74	1.44	0.89	5.94	1.01
27	1.61	0.93	2.79	0.76	7.55	2.05	1.60	0.98	1.91	0.77	6.49	1.50
28	2.03	0.52	1.30	0.86	7.81	1.04	2.65	0.86	1.41	0.33	6.77	1.08
29	2.03	0.58	1.67	0.82	9.21	1.96	3.79	0.79	1.79	0.67	6.81	2.65
30	0.90	0.76	2.14	0.40	9.73	0.97	2.36	0.79	2.61	0.81	7.01	2.00

Abbreviation: OED, organ equivalent doses; IMRT, intensity modulated radiotherapy; VMAT, volumetric modulated arc therapy; C-Breast, contralateral breast; I-Lung, ipsilateral lung; C-Lung, contralateral lung.

Table S5. EAR results for patients treated with IMRT/VMAT

	IMRT (EAR/10000 PY)						VMAT (EAR/10000 PY)					
No.	SC	Liver	C-Breast	Stomach	I-Lung	C-Lung	SC	Liver	C-Breast	Stomach	I-Lung	C-Lung
1	0.09	0.49	3.47	2.49	40.03	4.63	0.25	1.17	7.19	2.46	33.55	20.57
2	0.06	0.36	1.40	2.72	42.85	2.47	0.19	0.91	4.61	2.41	34.36	7.69
3	0.09	0.95	3.18	1.53	46.65	4.33	0.23	0.62	7.07	1.89	34.96	6.15
4	0.09	0.59	6.17	2.42	49.45	22.34	0.35	1.27	7.83	4.14	37.94	22.59
5	0.37	0.46	1.97	3.90	50.09	4.99	0.56	0.84	8.43	3.14	39.60	15.54
6	0.11	1.03	3.56	0.86	49.29	2.98	0.38	0.75	8.09	2.05	40.46	8.88
7	0.85	1.06	2.32	0.96	50.36	4.36	0.15	0.49	4.20	2.78	41.27	16.56
8	0.74	1.78	9.14	4.42	51.00	13.41	0.58	1.29	9.54	3.44	40.08	9.74
9	0.58	1.33	6.55	2.32	52.47	7.09	0.26	0.61	5.67	0.95	41.83	17.27
10	0.72	1.82	17.16	2.37	49.85	5.82	0.35	0.86	8.10	1.71	41.93	9.36
11	0.70	1.20	12.66	3.84	53.23	25.21	0.58	1.25	7.29	2.36	42.00	9.79
12	0.36	0.57	2.76	3.87	54.82	7.12	0.67	1.70	9.98	3.16	41.51	10.39
13	0.46	1.02	3.27	2.85	55.41	8.32	0.37	0.79	5.85	1.99	44.53	14.69
14	0.17	0.93	3.09	1.09	54.28	3.68	0.43	0.63	11.90	2.63	44.30	10.94
15	0.17	0.71	4.85	3.23	53.63	4.86	0.51	1.16	15.10	2.34	44.58	11.47
16	0.14	0.72	3.12	3.49	56.16	24.10	0.69	1.05	7.01	2.81	45.74	14.13
17	0.56	1.38	6.52	4.30	55.32	8.88	0.98	0.92	3.88	2.95	47.20	15.66
18	0.59	1.31	12.64	4.07	55.98	11.44	0.20	0.91	4.08	3.19	47.91	9.45
19	0.41	1.42	9.79	2.68	55.17	14.99	2.18	1.03	6.47	1.94	48.77	24.46
20	0.17	1.52	5.45	1.12	54.70	2.45	1.06	0.75	8.37	1.71	49.50	20.30
21	0.89	0.48	1.61	3.87	58.69	6.64	0.67	1.38	25.89	3.55	47.87	31.59
22	0.67	0.76	3.89	4.18	59.56	9.00	0.31	0.67	4.62	3.52	50.24	11.17
23	0.59	1.34	5.30	2.26	60.25	4.70	0.47	0.95	8.66	3.64	48.56	9.15
24	0.72	0.75	8.95	3.79	59.76	8.97	0.52	2.06	7.90	2.71	47.62	8.16
25	1.89	0.59	7.24	4.06	58.74	7.59	1.78	0.92	8.57	2.14	49.61	23.29
26	0.64	1.20	8.29	2.43	61.18	11.87	0.53	1.52	9.09	4.57	48.16	8.19
27	0.59	1.26	8.42	3.75	63.78	17.31	0.95	2.03	12.07	3.92	52.62	12.17
28	1.17	1.05	7.92	4.41	63.53	8.45	0.92	1.12	3.95	1.62	57.37	9.10
29	0.76	0.81	5.22	4.05	77.60	16.52	1.32	1.03	5.02	3.29	57.70	22.47
30	0.30	0.95	5.56	1.95	82.82	8.26	0.87	1.08	7.87	3.98	59.16	16.89

Abbreviation: EAR, excess absolute risks; IMRT, intensity modulated radiotherapy; VMAT, volumetric modulated arc therapy; C-Breast, contralateral breast; I-Lung, ipsilateral lung; C-Lung, contralateral lung. The EAR has units of excess cases per 10,000 person-years (PY)/Gy.

Table S6. Difference comparison between IMRT and VMAT with OED and EAR

Techniques / Organs	averaged OED (Gy) (30: 30)			averaged EAR (/10000 PY) (30: 30)		
	IMRT	VMAT	*p*	IMRT	VMAT	*P*
Spinal cord	1.22	1.54	0.27	0.52	0.64	0.28
C-Breast	1.48	2.03	<0.01	6.05	8.14	0.05
I-Lung	6.68	5.45	<0.01	55.89	45.36	<0.01
C-Lung	1.11	1.71	<0.01	9.43	14.26	<0.01
Liver	0.65	0.64	0.94	0.99	1.06	0.53
Stomach	0.60	0.55	0.38	2.98	2.77	0.42

Abbreviations: IMRT, intensity-modulated radiotherapy; VMAT, volumetric-modulated arc therapy; OED, organ equivalent doses; EAR, excess absolute risks; C-Breast, contralateral breast; I-Lung, ipsilateral lung; C-Lung, contralateral lung. The EAR has units of excess cases per 10,000 person-years (PY)/Gy.

Supplementary

Figures

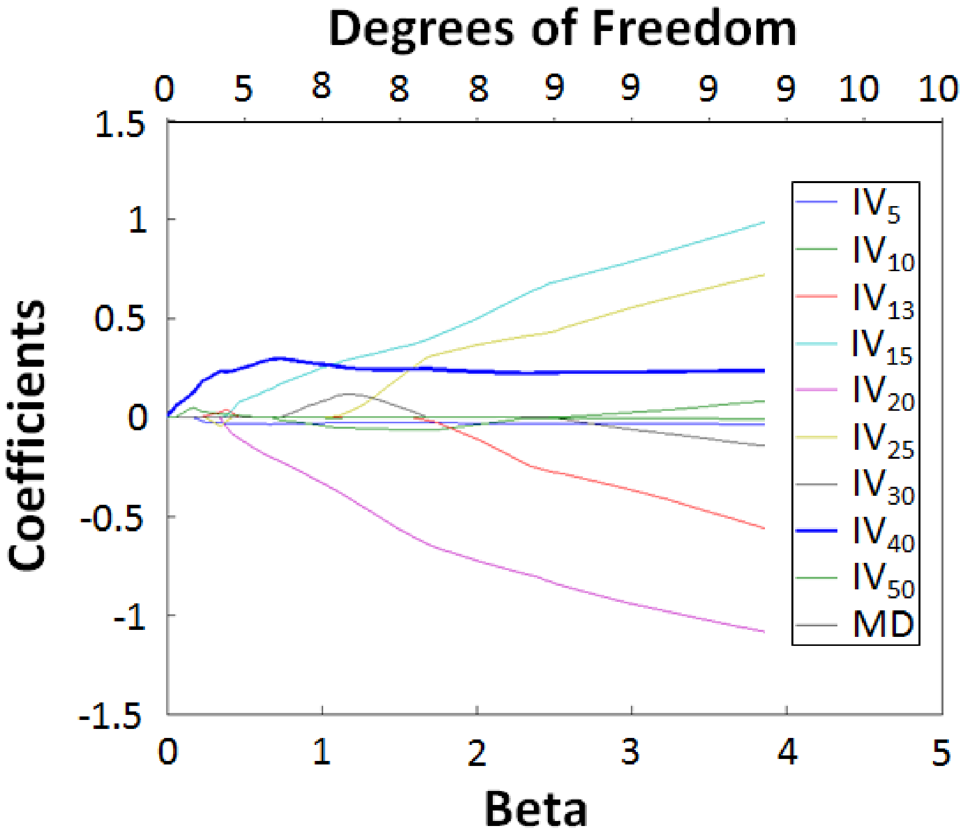

Factor ranking	Factor
**1**	**IV_{40}**
**2**	**IV_{50}**
**3**	**IV_{30}**
**4**	**IV_{25}**
**5**	**MD**
**6**	**IV_{20}**
**7**	**IV_{15}**
**8**	**IV_{13}**
**9**	**IV_{10}**
**10**	**IV_{5}**

Figure S1: The LASSO trace plot of dosimetric candidate predictive factors and the factors ranking list.

Abbreviations: IV_{40}: the volume of the ipsilateral lung receiving at least 40 Gy; the following parameters are similar; IV_{5}~IV_{50}; LASSO: least absolute shrinkage and selection operator.

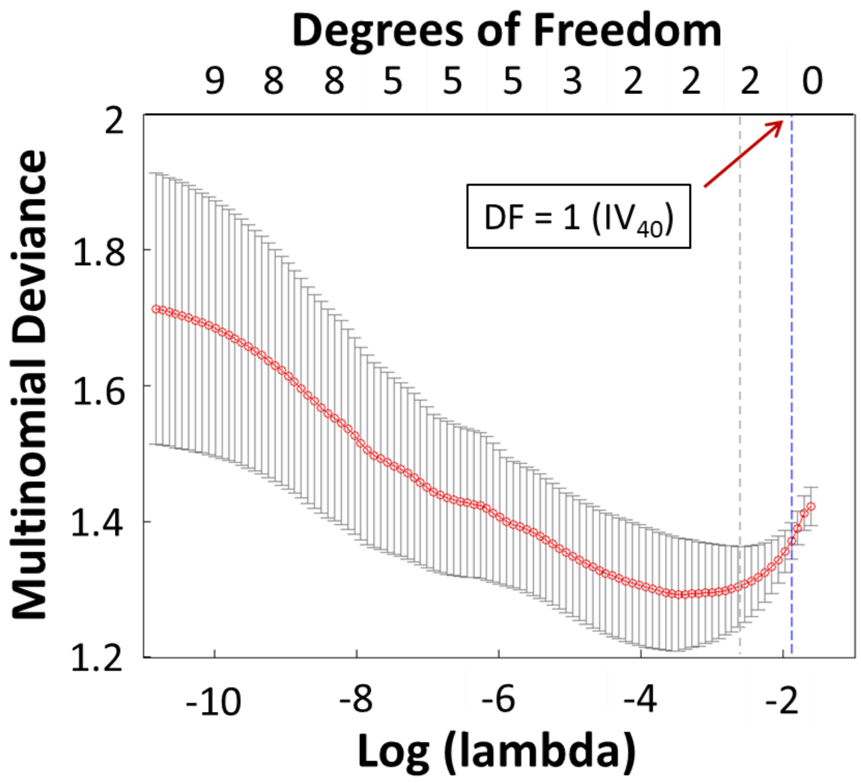

Figure S2: The cross-validated mean squared error (MSE) of the LASSOGLM fit. IV_{40} was selected as the dominant risk factor for the RP NTCP model by LASSO using cross-validation.

Abbreviations: LASSO, least absolute shrinkage and selection operator; RP, radiation-induced pneumonitis; NTCP, normal tissue complication probability; IV_{40}: the volume of the ipsilateral lung receiving at least 40 Gy;
